# Integrated 16S rRNA Sequencing and Metabolomics Reveals Niche-Specific Microbiome and Metabolome Changes Associated with *Toxoptera aurantii* Infestation

**DOI:** 10.3390/microorganisms14071463

**Published:** 2026-07-03

**Authors:** Yunchao Wang, Peipei Long, Nian Wen, Manting Zhang, Jingjing Li, Xiong Yan, Zhongjiu Xiao, Kun Yang

**Affiliations:** 1 College of Ecology, Zunyi Normal University, Zunyi 563006, China; wyc801suo@163.com (Y.W.); 18385328104@163.com (P.L.); 17885109904@163.com (N.W.); mmqcsj@163.com (M.Z.); ljj1314yyra@163.com (J.L.); yanxiongmail@163.com (X.Y.); xzj198099@163.com (Z.X.); 2 Key Laboratory of Integrated Pest Management on Crops in East China, Ministry of Agriculture and Rural Affairs, Nanjing Agricultural University, No. 1 Weigang, Nanjing 210095, China

**Keywords:** endosymbionts, metabolomics, microbiome, plant–insect interactions, tea plant

## Abstract

*Toxoptera aurantii* is a globally distributed piercing-sucking pest that severely threatens tea production. While the direct damage caused by aphid feeding is well documented, the systemic effects of infestation on plant-associated and soil microbial communities remain poorly understood. Here, we employed full-length 16S rRNA gene sequencing and untargeted metabolomics to investigate the influence of *T. aurantii* infestation on the microbiota of tea plants (*Camellia sinensis*) and rhizosphere soil across four sample compartments: aphid bodies, healthy leaves, aphid-infested leaves, and root-zone soil. Our results revealed pronounced niche-specific microbial assembly patterns. The aphid microbiome exhibited the lowest diversity and was dominated by obligate endosymbionts, including *Buchnera aphidicola* and the secondary symbiont *Serratia symbiotica*. Soil harbored the highest microbial diversity with a balanced phylum-level structure. Aphid infestation significantly reduced phyllosphere microbial diversity (Shannon index) and shifted community composition, with a decline in a sequence putatively assigned to *Methylobacterium brachiatum* and a modest increase in a taxon assigned to the opportunistic plant pathogen OTU assigned to *Dickeya chrysanthemi*. This pattern suggests a hypothesis that aphid infestation may create conditions permissive for such opportunistic pathogens, although experimental validation is required. Concurrently, infestation was associated with profound metabolic reprograming in tea leaves, including upregulation of defense-related flavonoids and terpenoids and downregulation of several primary metabolites. Notably, the phyllosphere of infested leaves showed reduced microbial diversity and an increased relative abundance of a 16S rRNA sequence assigned to *Dickeya chrysanthemi*, while certain plant-derived antimicrobial metabolites were decreased. These patterns suggest a possible association between aphid infestation, altered antimicrobial metabolite profiles and increased relative abundance of *Dickeya*-assigned sequences. These findings demonstrate that *T. aurantii* infestation triggers a systemic response in the aboveground compartments (aphid and leaf), while the soil compartment maintains a distinct and highly diverse microbial community that serves as a potential reservoir. The study characterizes microbial communities across these three compartments without inferring infestation-driven soil remodeling. This study advances our understanding of tripartite interactions in tea ecosystems and provides a basis for developing microbiome-based strategies for sustainable pest management.

## 1. Introduction

*Toxoptera aurantii* (Boyer de Fonscolombe) (Hemiptera: Aphididae) is a piercing-sucking pest that poses a serious threat to tea production worldwide [1]. In China, this aphid species is predominantly distributed in tea-growing regions south of the Yellow River, while globally it has spread across tropical and subtropical areas of Asia, Europe, Africa, America, and Australia [2]. Both adults and nymphs feed on phloem sap from tender shoots and young leaves, causing leaf curling, wrinkling, and hardening that severely inhibits shoot development and reduces tea yield [3,4]. Furthermore, the large quantity of honeydew excreted by *T. aurantii* contaminates lower leaves and readily induces sooty mold development, which obstructs photosynthesis and further weakens plant vigor [5]. Tea processed from infected buds and leaves exhibits reduced aroma, flat taste, and cloudy infusion color, substantially diminishing commercial value. Therefore, elucidating the mechanisms underlying tea aphid damage mechanisms and tea plant resistance represents a critical research priority for sustainable tea production.

As a specialized phloem-feeding insect, *T. aurantii* relies heavily on symbiotic microorganisms for survival and reproduction. The primary obligate endosymbiont *Buchnera. aphidicola* supplies essential amino acids deficient in phloem sap, enabling aphid survival on this nutritionally imbalanced diet [6,7]. Secondary symbionts play important roles in environmental adaptation and physiological regulation. For instance, *Serratia* spp. can enhance aphid thermotolerance by forming a complementary DNA mismatch repair system with *Buchnera* and stabilizing transcription of heat stress proteins [8]. Additionally, *Pseudomonas fluorescens* isolated from tea aphids exhibits pathogenic activity against other aphid species, suggesting potential roles in regulating natural populations [9]. As an oligophagous aphid, the symbiotic community structure of *T. aurantii* may differ from that of polyphagous aphids due to host plant chemical screening effects [10]. However, systematic marker-gene-based profiling of the symbiotic flora within the aphid–tea plant system remains lacking, particularly regarding how aphid infestation reshapes microbial communities across the plant–insect–soil continuum. Although the hypothesis of alginate in tea aphid honeydew has been shown to inhibit catechin synthesis and indirectly promote the proliferation of other herbivorous pests [11,12], whether symbiotic bacteria directly secrete effector proteins that modulate tea plant immune signaling pathways (e.g., JA or SA pathways) remains unknown. Current research has focused primarily on symbiont isolation and identification, leaving a critical gap in understanding the dynamic changes, interaction networks, and adaptive functions of the tea aphid microbiome.

Upon *T. aurantii* feeding, tea plants activate a multi-level defense response: (i) phytohormone signaling (JA and SA pathways), (ii) accumulation of secondary metabolites (flavonoids and terpenoids) as effector molecules, and (iii) activation of immune-related enzymes (PPO, POD, and SOD) as execution units [13]. The jasmonic acid (JA) signaling pathway plays a central role in tea plant defense against aphids. Aphid feeding induces expression of key JA pathway genes including CsLOX2.1/2.2/2.3 and *CsLOX3* (lipoxygenase genes) and, through regulatory modules involving CsMYC2 (a bHLH transcription factor) and CsWRKY23 (a WRKY transcription factor), drives synthesis of volatile terpenes such as β-elemene that directly interfere with aphid feeding behavior and enhance indirect resistance against other pests [11,14,15]. Concurrently, the salicylic acid (SA) pathway is significantly activated; exogenous SA application inhibits aphid population growth, associated with enhanced activities of polyphenol oxidase (PPO), peroxidase (POD), and superoxide dismutase (SOD). Among these enzymes, PPO and POD catalyze the oxidative polymerization of phenolics to form physical barriers. Concurrently, SA pathway enhances the activity of superoxide dismutase (SOD), which scavenges superoxide radicals and modulates ROS homeostasis, contributing to aphid feeding deterrence [16]. The tea plant defense response also involves dynamic accumulation of catechins regulated by the CsWRKY12-CsVQ4L module (a WRKY transcription factor and a VQ motif-containing protein), a process that influences honeydew composition and indirectly affects population dynamics of co-inhabiting pests [17]. Furthermore, insect feeding can reshape microbial community structure between leaves and roots, enriching bacteria such as *Alicyclobacillus* and *Streptomyces* that enhance microbial enzyme activities in soil and plant nutrient compensation capacity, forming a “plant-microbe-soil-insect” tripartite interaction network [18,19].

Although *T. aurantii* feeding primarily affects aboveground tissues, the induced physiological and metabolic responses may potentially influence rhizosphere microbiome structure and function through aboveground–belowground signaling linkages. However, as no uninfested soil control was collected in this study, we can only characterize the soil microbiome as a baseline compartment rather than establish causal remodeling effects. Activation of JA and SA pathways in infected leaves is accompanied by accumulation of β-elemene, flavonoids, and phenolic acids, which may be transmitted to the soil microenvironment through changes in root exudate composition [15,16]. Tea plant root exudates, rich in organic acids, sugars, and phenolics, can selectively enrich or inhibit specific rhizosphere microbial populations [20]. Evidence from other sucking pest–plant systems suggests that aphid feeding enhances rhizosphere microbial diversity and alters abundance of functional genes related to nitrogen cycling and organic acid metabolism [21]. Additionally, honeydew metabolites deposited on soil surfaces may act as exogenous carbon sources influencing rhizosphere microbial activity, forming a honeydew-root exudate–microbe regulatory network [22]. However, whether tea aphid-induced signaling molecules directly regulate rhizosphere microbial community assembly remains unclear, and causal relationships have not been established using sterile plant models.

In the present study, we employed full-length 16S rRNA gene sequencing (PacBio) and untargeted metabolomics (UPLC-MS/MS) to systematically investigate microbial communities across four ecological compartments (aphids, healthy leaves, infected leaves, and rhizosphere soil) under *T. aurantii* infestation conditions. We aimed to (i) characterize niche-specific microbial assembly patterns and identify key symbionts associated with *T. aurantii*; (ii) determine how aphid infestation alters phyllosphere microbial diversity and composition; (iii) profile metabolic reprograming in infected tea leaves; and (iv) characterize the soil microbial community as a distinct compartment to enable future investigations of aboveground–belowground linkages. Our integrated approach provides comprehensive insights into the tripartite interactions governing tea plant health and establishes a foundation for developing microbiome-based strategies for sustainable pest management.

## 2. Materials and Methods

### 2.1. Sample Collection

Samples of *T. aurantii*, tea leaves (*Camellia sinensis* (L.) O. Kuntze (Ericales: Theaceae)), and rhizosphere soil were collected from a commercial tea plantation located in Zunyi (27.42° N, 106.55° E), Guizhou Province, China, in September 2025. The tea cultivar was “Fuding Dabaicha”. The plantation had no pesticide application for the past 12 months. Four sample groups were established: aphid (Aphid), healthy leaf (Leaf), *T. aurantii*-infected leaf (Infected), and rhizosphere soil (Soil), each with four biological replicates.

Apterous adult aphids (approximately 50–100 individuals per sample) were collected from infected tea shoots and young leaves using sterile brushes, immediately transferred to sterile cryogenic vials, flash-frozen in liquid nitrogen, and stored at −80 °C until DNA extraction. From each replicate, 20 individuals were randomly selected for genomic DNA extraction. Healthy leaves (without visible damage or infestation) and infected leaves (exhibiting typical symptoms including curling, chlorosis, and honeydew deposition) were excised using sterile scissors, placed into sterile sampling bags, and transported to the laboratory on ice. Healthy and infected leaves were collected from different plants within the same tea plantation. This method recovers surface-attached bacteria but does not remove soluble honeydew or non-living DNA. Infected leaves exhibited typical symptoms (curling, chlorosis, and honeydew deposition) with a standardized damage level: at least 50 aphids per 10 cm^2^ leaf area and >30% of leaf surface showing chlorosis or curling. Leaves with no visible damage and zero aphids after careful inspection were designated as healthy. Upon arrival, leaf samples were processed to separately analyze epiphytic (surface-attached) microorganisms and leaf metabolites. To collect epiphytic microbiota, the leaf surface was gently swabbed with sterile cotton swabs pre-moistened with sterile water, followed by rinsing with 5 mL of sterile water. The resulting washing suspension was centrifuged, and the pellet was used for DNA extraction to target epiphytic microbial communities. This method inevitably loses some loosely attached or non-adherent microorganisms but effectively recovers the majority of surface-colonizing epiphytes. After removing surface microorganisms, the remaining leaf tissues were immediately processed for untargeted metabolomics analysis (UPLC-MS/MS), ensuring that detected metabolites originate from leaf tissues rather than surface contaminants. The remainder of the leaf tissues (not used for metabolomics) were not subjected to DNA extraction, as microbial DNA was obtained from the surface washing suspension as described above. After carefully removing the surface litter layer, rhizosphere soil adhering tightly to tea plant roots was collected from a depth of 0–20 cm. Soil from multiple points around each sampled plant was pooled to form one composite sample. Soil samples were passed through a 2 mm sterile sieve to remove stones, plant debris, and macrofauna, thoroughly mixed, divided into aliquots, flash-frozen in liquid nitrogen, and stored at −80 °C for DNA extraction. Because the tea plantation had widespread aphid infestation, the root-zone soil was collected from plants that exhibited visible aphid colonization on their shoots. No separate soil samples from entirely uninfested plants were collected, as such plants were not available in the plantation at the time of sampling. Therefore, the soil data represent a baseline characterization of the root-zone microbial community under infested field conditions, not a comparison between infested and uninfested states.

### 2.2. Genomic DNA Extraction

Total genomic DNA was extracted from all samples using the TIANGEN Genomic DNA Kit (Tiangen Biotech Co., Ltd., Beijing, China) following the manufacturer’s instructions. For aphid samples, approximately 20 randomly selected individuals per biological replicate were pooled for extraction. For leaf and soil samples, approximately 0.5 g of frozen powder was used. DNA integrity was assessed by electrophoresis on 1% (*w*/*v*) agarose gels. DNA concentration and purity (A260/A280 and A260/A230 ratios) were determined using a NanoDrop 2000 spectrophotometer (Thermo Fisher Scientific, Waltham, MA, USA). DNA stocks were stored at −20 °C until use.

### 2.3. Full-Length 16S rRNA Gene Amplification and PacBio Sequencing

The full-length bacterial 16S rRNA gene was amplified using the universal primer pair 27F (5′-AGRGTTTGATYNTGGCTCAG-3′) and 1492R (5′-TASGGHTACCTTGTTASGACTT-3′). DNA amplification, library construction, sequencing, and data analysis were performed by Biomarker Technologies Corporation (Beijing, China). PCR amplification was conducted in a 50 μL reaction volume containing 10 μL buffer, 0.2 μL Q5 High-Fidelity DNA Polymerase, 10 μL High GC Enhancer, 1 μL dNTP, 10 μM of each primer, and 60 ng genomic DNA. Thermal cycling conditions were: initial denaturation at 95 °C for 5 min; 15 cycles of 95 °C for 1 min, 50 °C for 1 min, and 72 °C for 1 min; and a final extension at 72 °C for 7 min. First-step PCR products were purified using VAHTS DNA Clean Beads. A second-round PCR was performed in a 40 μL reaction containing 20 μL 2× Phusion HF MM, 8 μL ddH_2_O, 10 μM of each primer, and 10 μL purified first-step PCR products. Thermal cycling conditions were: initial denaturation at 98 °C for 30 s; 10 cycles of 98 °C for 10 s, 65 °C for 30 s, and 72 °C for 30 s; and final extension at 72 °C for 5 min. All final PCR products were quantified using the Quant-iT dsDNA HS Reagent and pooled at equimolar concentrations. The amplified library was sequenced on the PacBio SMRT RS II DNA sequencing platform (Pacific Biosciences, Menlo Park, CA, USA).

### 2.4. Sequencing Data Processing and Bioinformatics Analysis

#### 2.4.1. 16S rRNA Data Processing

Raw sequencing reads were processed and demultiplexed using SMRT Link software (version 8.0) with parameters set to minPasses ≥5 and minPredictedAccuracy ≥0.9 to obtain circular consensus sequencing (CCS) reads. Subsequently, lima software (version 1.7.0) was employed to assign CCS sequences to corresponding samples based on their barcodes. CCS reads containing no primers and reads outside the expected length range (1200–1650 bp) were discarded through primer recognition and quality filtering using Cutadapt (version 2.7).

High-quality CCS reads were clustered into operational taxonomic units (OTUs) at 97% sequence similarity using USEARCH (v10.0). Representative sequences for each OTU were selected, and taxonomic assignment was performed using the RDP classifier (v2.2) against the SILVA database (release 138.1) with a minimum confidence threshold of 0.8. OTUs assigned to chloroplasts, mitochondria, or non-bacterial lineages were removed from downstream analyses. Species-level assignments are putative unless independently validated.

#### 2.4.2. Diversity Analyses

Alpha diversity metrics, including Shannon diversity index and Simpson diversity (1–D) index, were calculated using QIIME software (v1.8.0), where higher values indicate greater diversity. The Shapiro–Wilk test (SPSS 21.0) was performed to assess normality of alpha diversity indices. As data followed normal distribution, one-way analysis of variance (ANOVA) followed by Tukey’s Honestly Significant Difference (HSD) post hoc test was used to compare differences among sample groups. Beta diversity was evaluated using principal coordinate analysis (PCoA) based on Bray–Curtis distance matrices. Permutational multivariate analysis of variance (PERMANOVA) with 999 permutations was conducted to test for significant differences in community composition among groups. PCoA plots were generated using the ggplot2 package (v3.3.6) in R software (v4.1.2).

#### 2.4.3. Important Limitations Regarding Contamination and Low-Abundance Taxa

This study did not include field-relevant negative controls, nor did we perform source-tracking analyses. This is a serious limitation, especially for the low-biomass phyllosphere samples. Without these controls, we cannot exclude the possibility that some low-abundance OTUs, or even some species-level assignments, originated from reagent or environmental contamination during sampling, DNA extraction, or library preparation. Therefore, all species-level claims, particularly those based on low relative abundance (< 0.5% of the community in a given sample), are presented as putative assignments that require independent validation. The primary observations reported in this study—differences in alpha diversity, overall community composition (Bray–Curtis), and changes in dominant taxa—are robust to this limitation. However, inferences about low-abundance or unexpected taxa should be treated as hypothesis-generating.

### 2.5. Metabolite Extraction and UPLC-MS/MS Analysis

Frozen leaf samples were ground to a fine powder in liquid nitrogen. Approximately 100 mg of frozen powder was accurately weighed and transferred to a 2 mL microcentrifuge tube. Metabolites were extracted by adding 1.0 mL of pre-chilled (−20 °C) methanol:water (7:3, *v*/*v*) containing 10 μg/mL 2-chlorophenylalanine as internal standard. Samples were vortexed for 30 s, sonicated in an ice-water bath for 30 min, and incubated at −20 °C for 1 h to precipitate proteins. After centrifugation at 12,000× *g* for 15 min at 4 °C, supernatants were filtered through 0.22 μm PTFE syringe filters into LC-MS vials.

Untargeted metabolomics analysis was performed using a Vanquish UHPLC system coupled to a Q Exactive HF mass spectrometer (Thermo Fisher Scientific). Chromatographic separation was achieved on an ACQUITY UPLC HSS T3 column (2.1 × 100 mm, 1.8 μm particle size; Waters, Milford, MA, USA) maintained at 40 °C. The mobile phase comprised (A) 0.1% (*v*/*v*) formic acid in water and (B) 0.1% (*v*/*v*) formic acid in acetonitrile. The gradient elution program was: 0–2 min, 5% B; 2–4 min, 5–30% B; 4–8 min, 30–50% B; 8–10 min, 50–80% B; 10–12 min, 80–100% B; 12–14 min, 100% B; 14–14.1 min, 100–5% B; and 14.1–16 min, 5% B. Flow rate was 0.35 mL/min, and injection volume was 5 μL. Mass spectrometry was performed in both positive and negative electrospray ionization (ESI) modes with full scan range *m*/*z* 70–1050 at 70,000 resolution, followed by data-dependent MS/MS acquisition at 17,500 resolution.

Raw data were processed using Compound Discoverer software (v3.1; Thermo Fisher Scientific). Metabolites were identified by matching accurate mass, retention time, and MS/MS fragmentation patterns against public databases, including mzCloud, Human Metabolome Database (HMDB, version 5.0), MassBank, and KEGG (release 105.0). Metabolites with relative standard deviation (RSD) > 30% in quality control (QC) samples were filtered out.

Multivariate statistical analysis was performed using SIMCA software (version 17.0; Umetrics, Umeå, Sweden). Principal component analysis (PCA) was initially applied to visualize overall sample distribution and identify outliers. Orthogonal partial least squares discriminant analysis (OPLS-DA) was then conducted to maximize separation between Leaf and Infected groups. Model validity was assessed by seven-fold cross-validation and 200 permutation tests. Differential accumulated metabolites (DAMs) between healthy and infested leaves were identified using univariate statistics only. A metabolite was considered significantly differential if it met all of the following criteria: (1) |log_2_(fold change)| > 1 (i.e., >2-fold upregulation or <0.5-fold downregulation), (2) *p* < 0.05 from Student’s t-test, and (3) Benjamini–Hochberg false discovery rate (FDR) corrected q < 0.05. Orthogonal partial least squares discriminant analysis (OPLS-DA) was performed only as an exploratory visualization to assess sample separation. However, because the model exhibited very low predictive ability (Q^2^(cum) = 0.0945) and possible overfitting (permutation test), VIP values were not used as a selection criterion for DAMs.

Fold change (FC) was calculated as the ratio of mean peak area in Infected group to that in Leaf group; log_2_FC > 1 or <−1 indicated upregulation or downregulation, respectively. Quality control (QC) samples were injected every 10 injections throughout the run; their clustering in PCA was used to assess instrument stability. KEGG pathway enrichment analysis of differentially accumulated metabolites was performed using the MetaboAnalyst web server (version 5.0).

## 3. Results

### 3.1. Overview of Full-Length 16S rRNA Gene Sequencing and Taxonomic Profile

Full-length 16S rRNA gene sequencing was performed to characterize the bacterial communities associated with *T. aurantii* and its surrounding environment. After quality filtering, a total of 175,266 circular consensus sequences (CCS) were obtained across all samples, with an average effective rate of 95.8% and an average read length of 1448 bp (Table 1).

The taxonomic composition was annotated from phylum to species level. The soil samples exhibited the highest microbial richness, with up to 18 phyla, 37 classes, 101 orders, 172 families, 268 genera, and 331 species detected in a single soil sample (soil-1). In contrast, aphid-associated communities were the least diverse, with a maximum of 8 phyla, 13 classes, 30 orders, 47 families, 63 genera, and 70 species in a single aphid sample (aphid-2).

Notably, the bacterial richness in infested leaves (post-aphid feeding) was intermediate between that of healthy leaves and aphids. For instance, infested leaf-2 contained 13 phyla, 20 classes, 43 orders, 70 families, 106 genera, and 130 species, whereas healthy leaf-4 contained 14 phyla, 19 classes, 46 orders, 79 families, 133 genera, and 191 species. This pattern suggests that aphid infestation may reduce the complexity of the leaf-associated bacterial community.

At the overall dataset level (all samples combined), a total of 22 phyla, 45 classes, 119 orders, 220 families, 380 genera, and 521 species were identified, highlighting the substantial microbial diversity across different niches in this system.

Principal coordinate analysis (PCoA) based on Bray–Curtis distances revealed distinct clustering patterns among sample groups. PERMANOVA confirmed significant differences in community composition (R^2^ = 0.896, *p* < 0.01). Soil samples formed a separate cluster, clearly apart from all aboveground samples (aphids, healthy leaves, and infected leaves), while the three aboveground groups clustered apart from each other (Figure S1).

Rarefaction analysis demonstrated that all four sample groups (aphid, healthy leaf, infected leaf, and soil) reached clear plateaus at their respective maximum sequencing depths—aphids at 2000–3000 reads (OTUs: 30–80), leaf groups at 5000–8000 reads (healthy: 150–230; infected: ~50–160), and soil at 6000–8000 reads (OTUs: ~380–600)—confirming that sequencing depth was sufficient for comparative analysis of microbial diversity across ecological niches (Figure S2).

### 3.2. Dominant Microbial Taxa at the Phylum and Species Levels Across Different Sample Groups

**Figure 1:**
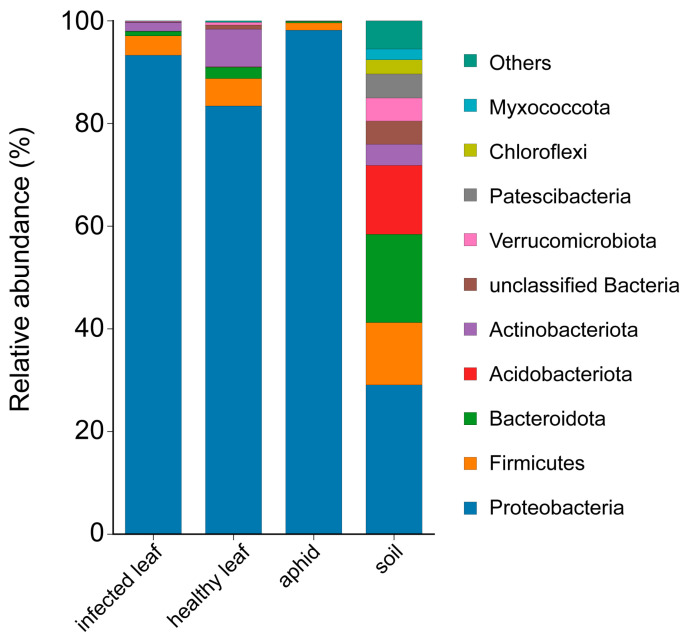
Relative abundance of bacterial communities at the phylum level associated with *Toxoptera aurantii*, healthy and infected tea leaves, and rhizosphere soil.

The bacterial community composition was characterized at both the phylum and species levels (Table 1). At the phylum level, a strong niche-specific pattern was observed. Proteobacteria was overwhelmingly dominant in aphid-associated communities (93.3%) and healthy leaves (98.2%) and remained the most abundant phylum in infected leaves (83.4%). In stark contrast, the soil microbiome exhibited a significantly more balanced and diverse phylum structure. Proteobacteria constituted only 29.1% of the soil community, which was co-dominated by Bacteroidota (17.2%), Acidobacteriota (13.4%), Firmicutes (12.1%), and several other phyla (e.g., Patescibacteria and Chloroflexi) that were nearly absent or present at very low levels in the aboveground samples (aphids and leaves) (Figure 1). We emphasize that these are putative species-level assignments based on full-length 16S rRNA similarity; definitive species identification would require additional methods.

**Figure 2:**
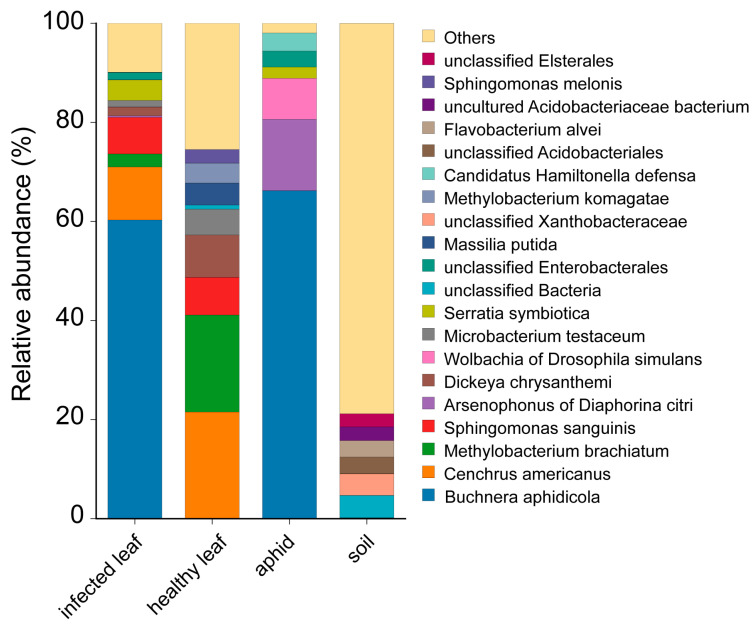
Relative abundance of bacterial communities at the species level associated with *Toxoptera aurantii*, healthy and infected tea leaves, and rhizosphere soil.

At the species level, the distinct ecological niches were further delineated by key taxonomic markers. Aphids and healthy leaves shared a high relative abundance of obligate endosymbionts, primarily Buchnera aphidicola (60.3% in aphids and 66.2% in healthy leaves) and *Arsenophonus* endosymbiont (0.31% in aphids and 14.4% in healthy leaves). The aphid microbiome was uniquely characterized by the presence of the secondary endosymbiont Serratia symbiotica (4.1%) (Figure 2).

Aphid infestation was associated with changes in the leaf microbiota. Compared to healthy leaves, infested leaves showed a modest increase in the relative abundance of an OTU assigned to *Dickeya chrysanthemi* (from 8.74% to 9.74%) and a decrease in *Methylobacterium brachiatum*. The soil harbored a complex community with no single dominant species; its composition was characterized by a high proportion of unclassified bacteria and uncultured taxa from groups like Acidobacteriales and Xanthobacteraceae, along with species such as Flavobacterium alvei (3.3%), which were unique to this habitat (Figure 2).

LEfSe analysis (LDA threshold = 4.0, Wilcoxon rank-sum test, α = 0.05) was performed to identify statistically significant differentially abundant taxa between healthy and infested leaves. Due to the limited sample size (n = 4 per group) and the lack of inherent multiple-testing correction in LEfSe, these results should be considered provisional and require independent validation. Compared to healthy leaves, the microbiota of infested leaves was characterized by a significant enrichment of the opportunistic pathogen *Dickeya chrysanthemi* (LDA = 4.62, *p* = 0.0074), *Serratia symbiotica* (LDA = 4.84, *p* = 0.0048), and *Sphingomonas sanguinis* (LDA = 4.89, *p* = 0.0048). Conversely, healthy leaves were significantly enriched with *Methylobacterium brachiatum* (LDA = 4.99, *p* = 0.0147), *Massilia putida* (LDA = 4.44, *p* = 0.0206), and *Microbacterium testaceum* (LDA = 4.52, *p* = 0.0449) (Table S1). This assignment is tentative; we do not claim confirmed presence or pathogenicity of *D. chrysanthemi* based on 16S data alone.

### 3.3. Statistical Analysis of Alpha Diversity Indices Across Different Sample Groups

**Figure 3:**
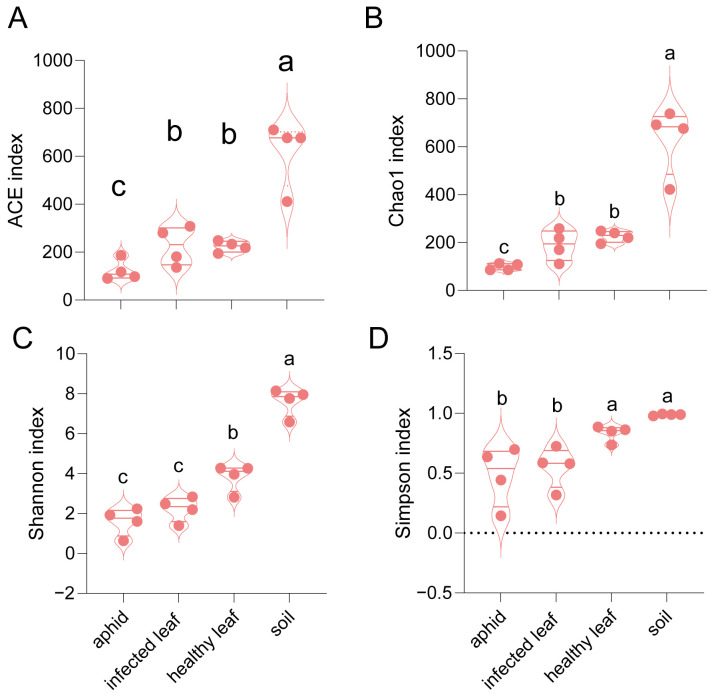
Alpha diversity indexes of bacterial communities at the species level associated with *Toxoptera aurantii*, healthy and infected tea leaves, and rhizosphere soil, including ACE index (**A**), Chao1 index (**B**), Shannon index (**C**) and Simpson index (**D**). Different letters mean significant differences between different groups by one-way analysis of variance (ANOVA) followed by Tukey’s Honestly Significant Difference.

To assess the differences in microbial alpha diversity across the four sample groups (Aphid, Infected Leaf, Soil, and Healthy Leaf), one-way analysis of variance (ANOVA) was performed on four key indices: ACE and Chao1 (richness estimators) and Simpson (1-D) and Shannon (diversity indices). The results revealed highly significant differences among the groups for all indices (Figure 3). Homogeneity of multivariate dispersions was confirmed using betadisper followed by permutest (999 permutations). The test was not significant (F = 1.845, *p* = 0.146), confirming that the assumptions for PERMANOVA were met. Therefore, the significant PERMANOVA result (R^2^ = 0.896, *p* < 0.01) reflects genuine differences in community composition (centroids) rather than differences in within-group dispersion.

Post hoc pairwise comparisons using Tukey’s Honestly Significant Difference (HSD) test were conducted to delineate the specific inter-group differences (Figure 3). The Soil group harbored a significantly richer and more diverse community than all other groups, as evidenced by its highest Shannon and Simpson indices (all *p* < 0.001 vs. Aphid, Infected Leaf, and Healthy Leaf). The aphid-associated microbiota was the least rich, with significantly lower Shannon index compared to both Healthy and Infected Leaf groups (all *p* < 0.01).

The Infected Leaf group exhibited a significantly compromised microbial diversity compared to the Healthy Leaf group. This was supported by a significantly lower Shannon index (*p* < 0.001). This result indicates that aphid herbivory primarily reduced community evenness and overall diversity rather than the sheer number of species.

### 3.4. Differential Metabolite Analysis Between Healthy and Infested Leaves

Based on the non-targeted metabolomics analysis comparing tea leaves infected by aphids (INFECT group) with uninfected control leaves (CK group), a profound reprograming of the leaf metabolome was observed. To assess data quality and instrument stability, quality control (QC) samples were injected every 10 runs. The median correlation coefficient among QC samples was 0.994 (above the recommended threshold of 0.8), and the relative standard deviation (RSD) of metabolite intensities was below 0.6 for 95.9% of all detected features (QC_RSD_percent = 0.959). These metrics indicate excellent instrument stability and reproducibility throughout the analytical batch. Furthermore, the inter-sample correlation matrix showed high intra-group correlations: healthy leaf replicates (CK1–CK6) had Pearson correlation coefficients ranging from 0.78 to 0.94 and infected leaf replicates (INFECT1–INFECT6) ranged from 0.74 to 0.96. In contrast, correlations between healthy and infested samples were generally lower (ranging from 0.74 to 0.92), suggesting that the metabolic difference induced by aphid infestation is greater than the biological variation within each group. This supports the robustness of subsequent multivariate analyses (Figure S3).

OPLS-DA was performed to compare the metabolic profiles of tea leaves infested by *T. aurantii* (INFECT) and uninfested controls (CK). The model included one predictive component (pre = 1) and two orthogonal components (ort = 2). The cumulative explained variation in X (R^2^X(cum)) was 0.418, and the cumulative explained variation in Y (R^2^Y(cum)) was 0.983, indicating an excellent fit to the training data. However, the cumulative predictive ability (Q^2^(cum)) was only 0.0945, suggesting that the model has limited cross-validated predictive power. Permutation testing gave an intercept for Q^2^ of 0.033 and a slope of 0.061, further supporting the possibility of model overfitting (Figure S4).

Principal component analysis (PCA) was performed on the metabolite profiles of healthy (CK, n = 6) and *T. aurantii*-infested (INFECT, n = 6) tea leaves. The first two principal components (PC1 and PC2) explained [23.59%] and [20.52%] of the total variance, respectively. Despite some overlap, the INFECT samples tended to separate from CK along both PC1 and PC2, indicating that aphid infestation induced substantial metabolic reprograming (Figure S5).

**Figure 4:**
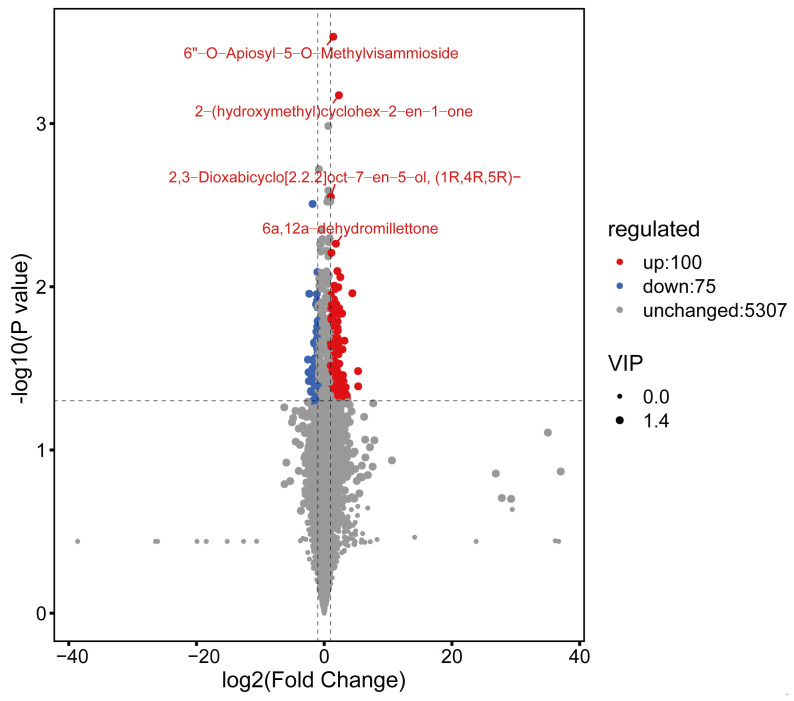
Volcano plot showing differentially accumulated metabolites between healthy and *Toxoptera aurantii*-infected tea leaves.

Statistical analysis using |log_2_FC| > 1 and FDR-corrected q < 0.05 (no VIP threshold) identified 175 significantly differentially accumulated metabolites (100 upregulated and 75 downregulated in infested leaves relative to healthy controls; see Table 2). Notably, a widespread downregulation of various primary and specialized metabolites was detected. This included a reduction in amino acids and derivatives such as N-Acetyl-D-phenylalanine (log_2_FC = −2.09), organic acids like 2-Hydroxy-4-[[[(4-methylphenyl)sulfonyl]oxy]amino]benzoic acid acetyl (log_2_FC = −2.54), sugars such as D-Xylulose (log_2_FC = −1.04) and fatty alcohols. The downregulation of key metabolites including several phenolic acids and a plant-derived alkaloid further suggests a disruption in primary metabolism (Figure 4, Table 2 and Table S1).

In contrast, a significant upregulation of numerous secondary metabolites, particularly those associated with plant defense, was triggered by aphid feeding. This induction was putatively annotated flavonoids/terpenoids biosynthesis pathways. Key flavonoids, including (+)-Gallocatechin (log_2_FC = 1.14), Pinocembrin (log_2_FC = 1.25), and Xanthohumol B (log_2_FC = 1.65), were markedly accumulated. Substantial increases were also observed in various terpenoids (e.g., Valeriandoid A, log_2_FC = 1.62), alkaloids, phenolic compounds, and coumarins. The accumulation of these defense-related compounds, such as Hydroquinone (log_2_FC = 1.56) and Cyanidin-3-O-(6″-O-malonyl-2″-O-glucuronyl) glucoside (log_2_FC = 1.93), indicates a targeted activation of specific biosynthetic pathways in response to aphid herbivory (Table S1).

## 4. Discussion

The intricate interplay between herbivorous insects, their host plants, and the associated microbial communities constitutes a fundamental axis of ecological and evolutionary dynamics. This study employed full-length 16S rRNA gene sequencing and untargeted metabolomics to systematically delineate the influence of *T. aurantii* infestation on the microbiota of tea plants (*C. sinensis*) and the surrounding soil. Our results reveal correlative patterns of niche-specific microbial assembly and plant metabolic changes, offering correlative insights into tripartite interactions within this system.

Our findings demonstrate a high degree of niche-specific microbial assembly. The aphid-associated microbiome exhibited the lowest alpha diversity and was overwhelmingly dominated by *Proteobacteria* (>93%), characterized by a consortium of known insect symbionts. The prevalence of *B. aphidicola* (60.3%) aligns with its well-established role as the primary obligate endosymbiont, provisioning essential amino acids deficient in phloem sap [6,7]. The co-occurrence of *Arsenophonus* and, uniquely, *Serratia symbiotica* (4.1%) in aphids is particularly noteworthy. The presence of *Serratia* corroborates recent findings by [8], who demonstrated that this secondary symbiont can enhance aphid thermotolerance through a complementary DNA mismatch repair system with *Buchnera* and stabilize transcription of the small heat stress protein IbpA. These observations suggest that the *T. aurantii* microbiome may be a dynamic entity that could bolster host adaptation to environmental stresses. The absence of these specialized symbionts in other niches, especially soil, underscores the strict host dependency and vertical transmission of these bacteria, thereby creating a highly specialized microbial hub within the aphid.

Concurrently, infected leaves became enriched with several bacterial taxa. Among these, *Methylobacterium brachiatum* and *Sphingomonas melonis* are considered potentially plant-beneficial bacteria, known for their plant growth-promoting or xenobiotic-degrading abilities [15]. In contrast, the well-known phytopathogen *Dickeya chrysanthemi* also increased substantially (from 8.74% in healthy leaves to 9.74% in infected leaves). The increased relative abundance of a 16S rRNA sequence assigned to *Dickeya chrysanthemi* in infested leaves is correlative and does not imply causation. One hypothesis consistent with these data—but not yet tested—is that aphid infestation, together with the observed downregulation of certain plant-derived antimicrobial metabolites, could alter the phyllosphere environment in a manner that favors the relative proliferation of such opportunistic taxa [16]. We emphasize that we did not isolate *Dickeya* strains, perform pathogenicity assays, quantify antimicrobial activity directly, or validate via qPCR. Therefore, this interpretation remains a hypothesis that requires direct experimental testing.

In stark contrast to aboveground niches, the soil microbiome exhibited the highest diversity and a balanced phylum-level structure, co-dominated by *Proteobacteria*, *Bacteroidota*, *Acidobacteriota*, and *Firmicutes*. This immense diversity reflects the complex and heterogeneous nature of the soil environment, serving as a robust microbial reservoir. The presence of taxa like *Acidobacteriota* and *Chloroflexi* indicates a community adapted to oligotrophic conditions and complex carbon cycling [18,19]. Although we did not directly measure soil enzyme activities or nutrient-cycling genes, the prevalence of these phyla suggests that rhizosphere soil maintains a stable community core responsible for fundamental ecosystem functions. Detection of species like *Flavobacterium alvei*, involved in organic matter decomposition, further supports this functional perspective. Our study did not include a direct comparison between soil from infested versus uninfected plants; therefore, we cannot assess whether aboveground herbivory influences the soil microbiome. The soil data presented here serve as a baseline characterization of the root-zone bacterial community under the field conditions at the time of sampling. The high diversity and balanced phylum-level structure (co-dominated by Proteobacteria, Acidobacteriota, and Firmicutes) indicate that the root-zone soil acts as a robust microbial reservoir. Future studies with paired rhizosphere samples from infested and uninfected plants are needed to test for potential aboveground–belowground effects.

The integration of our 16S rRNA and metabolomics data paints a holistic picture of the plant’s response to *T. aurantii*. The plant activates a direct chemical defense through accumulation of toxic and deterrent secondary metabolites [11,17]; infestation was associated with shifts in phyllosphere community structure. The downregulation of several plant-derived antimicrobial metabolites (e.g., certain coumarins and phenolic glycosides, log_2_FC < −1.5) together with a modest increase in the relative abundance of an OTU assigned to the opportunistic plant pathogen *Dickeya chrysanthemi* is consistent with the hypothesis that aphid infestation compromises the plant’s basal immunity. Rather than a strategic reallocation of resources, this pattern likely reflects a defense cost; the strong activation of herbivore-specific secondary metabolism may come at the expense of maintaining a robust antimicrobial barrier, thereby increasing susceptibility to secondary infections. This interpretation aligns with the observed reduction in phyllosphere microbial diversity (Shannon index) and the increased relative abundance of a *Dickeya*-assigned OTU. This trade-off creates a niche for specialized, resilient bacteria that may, in turn, offer benefits to the stressed plant. For instance, some *Methylobacterium* species are known plant growth promoters and can produce vitamins, potentially aiding plant recovery from herbivory damage. This emerging model, in which the plant immune system (JA/SA pathways) orchestrates not only its own chemical arsenal but also the recruitment or suppression of its microbial partners, represents a frontier in understanding plant health and resilience [14].

The distinct microbial signatures in aphids, leaves, and soil observed here provide a correlative description of three compartments under field conditions with aphid infestation. We cannot draw any conclusions about aboveground–belowground signaling because we did not analyze root exudates, root metabolomes, or soil from uninfested control plants. Nevertheless, the profound metabolic changes in the leaf—particularly the upregulation of flavonoids and terpenoids—generate a testable hypothesis for future research: that some of these compounds, if transported to roots and exuded into the rhizosphere, could, in principle. influence soil microbial communities. We emphasize that this remains entirely speculative based on the current data. As a future hypothesis, we propose that flavonoids and other aphid-induced chemical changes in the leaf could serve as long-distance signals that may eventually reshape the rhizosphere microbial community. Testing this hypothesis will require a coupled experimental design that simultaneously monitors aphid infestation, leaf and root metabolomes, root exudate profiles, and rhizosphere metagenomes to directly trace this systemic signaling cascade.

A key limitation is the absence of field-relevant negative controls (e.g., extraction/PCR blanks, mock communities, and source-tracking). Although laboratory blanks showed no amplification, they cannot exclude contamination from field collection or complex matrices [23]. Without source-tracking or mock communities, we cannot assess cross-sample contamination or false-positive taxonomy [24]. Thus, results represent a conservative view of resident communities. Notably, such control omissions are common in plant microbiome studies (only 30% used negative controls and 10% positive controls) [25]. Future studies should include appropriate controls and source-tracking.

## 5. Conclusions

This study provides correlative evidence that *T. aurantii* infestation is associated with distinct microbial community patterns across aphids, tea leaves, and root-zone soil. The phyllosphere microbiome of infested leaves showed reduced diversity (Shannon index) and a modest increase in the relative abundance of an OTU assigned to the opportunistic plant pathogen *Dickeya chrysanthemi*. Metabolomic analysis revealed upregulation of anti-herbivore secondary metabolites coincident with downregulation of several plant-derived antimicrobial compounds. The defense trade-off hypothesis, which posits that activation of herbivore-specific metabolism may come at the expense of antimicrobial defense and thereby increase susceptibility to opportunistic pathogens, is consistent with these patterns but requires functional validation. However, all findings are correlative, and causal relationships remain to be experimentally validated. The soil data are presented as a baseline characterization of the root-zone microbial compartment; no inference about infestation-driven soil remodeling can be drawn due to the absence of uninfested controls. This study offers a nuanced, hypothesis-generating perspective on plant–insect–microbe interactions and highlights the need for functional validation before translating these findings into sustainable pest management strategies.

## Figures and Tables

**Table 1:** Summary of full-length 16S rRNA gene sequencing data for *Toxoptera aurantii*, tea plant (*Camellia sinensis*) leaf, and soil samples.

Sample	Raw CCS	Clean CCS	Effective CCS	AvgLen (bp)	Effective (%)	Genus	Species
aphid-1	11,794	11,791	11,569	1468	98.09	45	48
aphid-2	12,250	12,247	10,839	1459	88.48	63	70
aphid-3	13,016	13,015	12,858	1470	98.79	41	45
aphid-4	12,042	12,040	11,468	1468	95.23	47	52
infected leaf-1	11,185	11,175	11,041	1458	98.71	104	126
infected leaf-2	10,112	10,100	9786	1440	96.78	105	129
infected leaf-3	8103	8101	8085	1468	99.78	55	67
infected leaf-4	14,362	14,358	13,745	1454	95.70	89	108
soil-1	13,378	13,352	12,415	1447	92.80	268	330
soil-2	13,538	13,531	12,836	1444	94.81	252	305
soil-3	12,665	12,656	12,450	1457	98.30	189	243
soil-4	12,820	12,817	11,510	1441	89.78	246	310
leaf-1	12,653	12,653	11,753	1430	92.89	102	143
leaf-2	12,227	12,226	11,819	1424	96.66	95	122
leaf-3	12,384	12,382	12,002	1422	96.92	118	157
leaf-4	10,971	10,968	10,818	1429	98.61	133	190

Footnote: Values represent four biological replicates per group: aphid-1 to aphid-4 (aphid bodies), leaf-1 to leaf-4 (healthy leaves), infected leaf-1 to infected leaf-4 (aphid-infested leaves), soil-1 to soil-4 (root-zone soil). The sample “aphid-2” is used as an example in the main text (Section 3.1). Replicate numbers indicate independent biological replicates, not technical replicates.

**Table 2:** Summary of differentially accumulated metabolites in tea leaves infected by *Toxoptera aurantii* compared to healthy controls.

Metabolic Class	Up-Regulated (No.)	Down-Regulated (No.)	Representative Metabolite (log_2_FC, KEGG Pathway)
Flavonoids (incl. flavanols, anthocyanins, chalcones)	18	3	(+)-Gallocatechin (1.14, ko00941), Pinocembrin (1.25, ko00941), Xanthohumol B (1.65)
Terpenoids (mono-, di-, sesqui-, tri-, iridoids)	14	4	Valeriandoid A (1.62), rosiridoside A (1.31), 14-deoxy-14α,15α-epoxyponasteroside A (−1.79)
Phenylpropanoids/Coumarins	6	2	esculin 6′-gallate (1.44), 6-Methoxymellein (1.06), 1H-2-Benzopyran-1-one,8-hydroxy-3-(hydroxymethyl)-6-methoxy- (−1.16)
Alkaloids	3	2	Triptersinine C (−1.39), 1-Desacetylwilforgine (1.60)
Phenolic acids/Polyphenols	12	8	Hydroquinone (1.56), Papuabalanol A (1.12), Benzoic acid, 3,4,5-trihydroxy-, 2,3-dihydroxypropyl ester (−1.34)
Amino acids & derivatives	2	15	N-Acetyl-D-phenylalanine (−2.09, ko00470), Dihydro-rhizobitoxin (−1.15)
Organic acids (non-phenolic)	6	9	1′-decarboxydehydrodigallic acid (1.05), 2-Hydroxy-4-[[[(4-methylphenyl)sulfonyl]oxy]amino]benzoic acid acetyl (−2.54)
Sugars & glycosides	7	6	D-Xylulose (−1.04, ko00040), UDP-kanosamine (1.19, ko00524)
Lipids (fatty acyls, steroids, isoprene lipids)	8	11	LysoPE 16:1 (1.72), crassumolide D (−1.16)
Nucleotides & derivatives	5	3	N-Isobutyrylguanosine (1.74), 2-Phenylaminoadenosine (1.33), [(2R,3S,4R,5R)-5-(2-amino-6-oxo-1H-purin-9-yl)-3,4-dihydroxyoxolan-2-yl]methyl phosphate (−1.02)
Others/unclassified	19	12	teasperin (5.30), bancroftinone (3.36), 2-Hydroxy-4-[[[(4-methylphenyl)sulfonyl]oxy]amino]benzoic acid acetyl (−2.54)
Total	100	75	Full list provided in Supplementary Table S1

Notes: Differentially accumulated metabolites were identified using |log_2_FC| > 1 and FDR-corrected q < 0.05. OPLS-DA VIP was not used as a selection criterion due to low model predictivity (Q^2^(cum) = 0.0945). All annotations are putative (MSI levels 2–3).

## Data Availability

The raw 16S rRNA gene sequencing reads generated in this study have been deposited in the NCBI Sequence Read Archive (SRA) under BioProject accession number PRJNA1419526. The untargeted metabolomics raw data and processed files have been deposited in the MetaboLights repository with the validate number MTBLS14697. Further inquiries can be directed to the corresponding authors.

## Supplementary Materials

The following supporting information can be downloaded at: https://www.mdpi.com/article/10.3390/microorganisms14071463/s1, Figure S1. Principal coordinate analysis of *Toxoptera aurantia*, host plant and soil. Figure S2. Rarefaction curves of bacterial communities across the four sample groups. Figure S3. Pearson correlation heatmap of metabolite profiles between healthy (CK) and *T. aurantii*-infested (INFECT) tea leaf samples. Figure S4. OPLS-DA model components and cumulative explained variances for the comparison between healthy (CK) and *T. aurantii*-infested (INFECT) tea leaves. Figure S5. PCA score plot of metabolite profiles from healthy (CK) and *T. aurantii*-infested (INFECT) tea leaves. Table S1. Significantly enriched bacterial taxa in each sample group identified by LEfSe analysis (LDA score > 4, Wilcoxon rank-sum test, *p* < 0.05).
