# Supplementary material for: Integrated 16S rRNA Sequencing and Metabolomics Reveals Niche-Specific Microbiome and Metabolome Changes Associated with Toxoptera aurantii Infestation
Source: Microorganisms. 2026 Jul 3;14(7):1463. doi: 10.3390/microorganisms14071463 (PMC13413915; doi:10.3390/microorganisms14071463)
Supplement: Supplementary file 1 [file microorganisms-14-01463-s001.zip › microorganisms-4291299-supplementary.pdf]

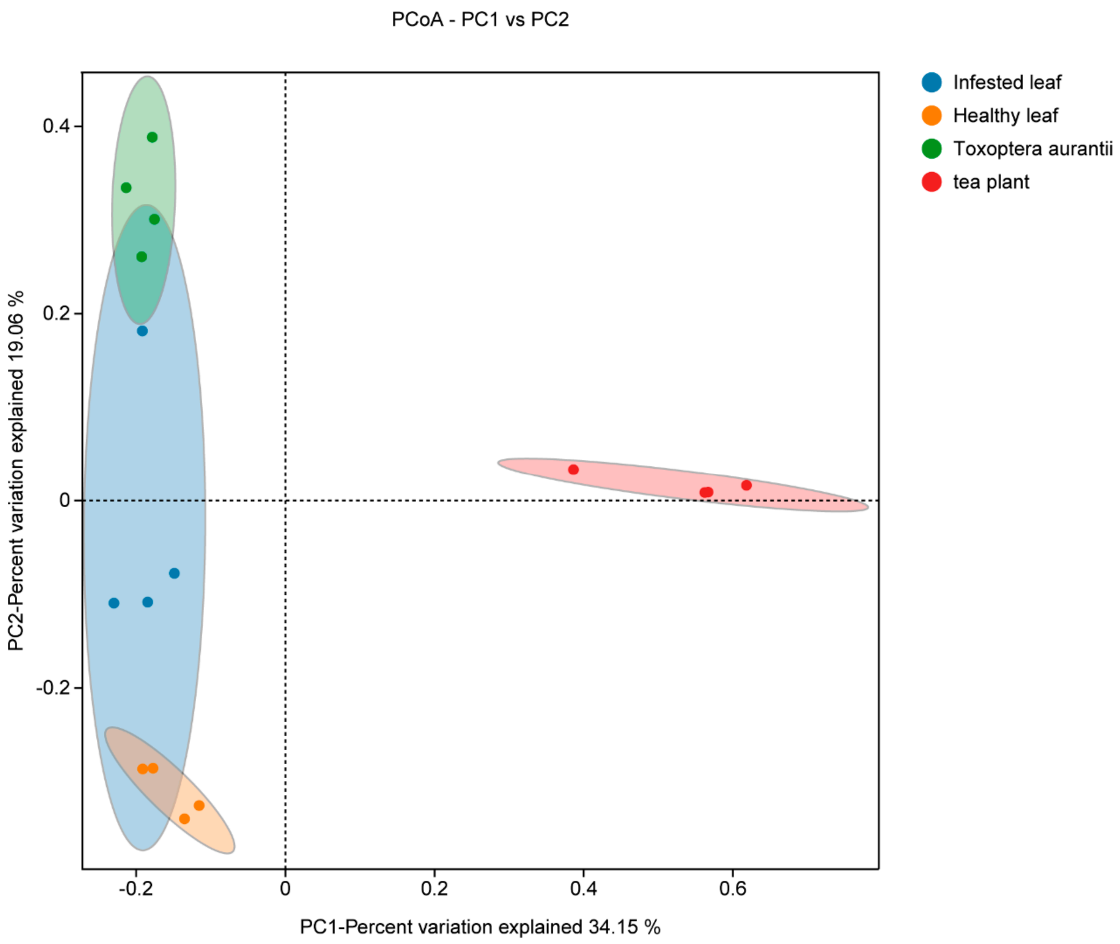

Figure S1. Principal coordinate analysis of *Toxoptera aurantia*, host plant and soil.

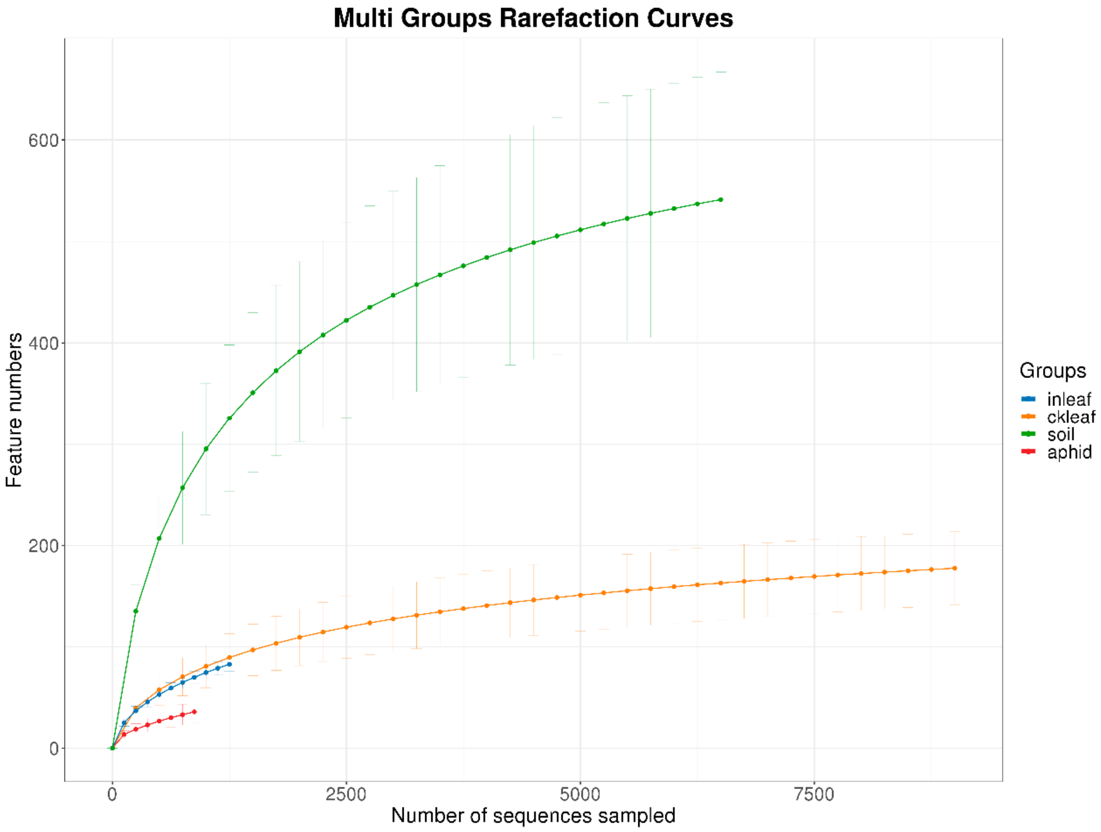

Figure S2. Rarefaction curves of bacterial communities across the four sample groups.

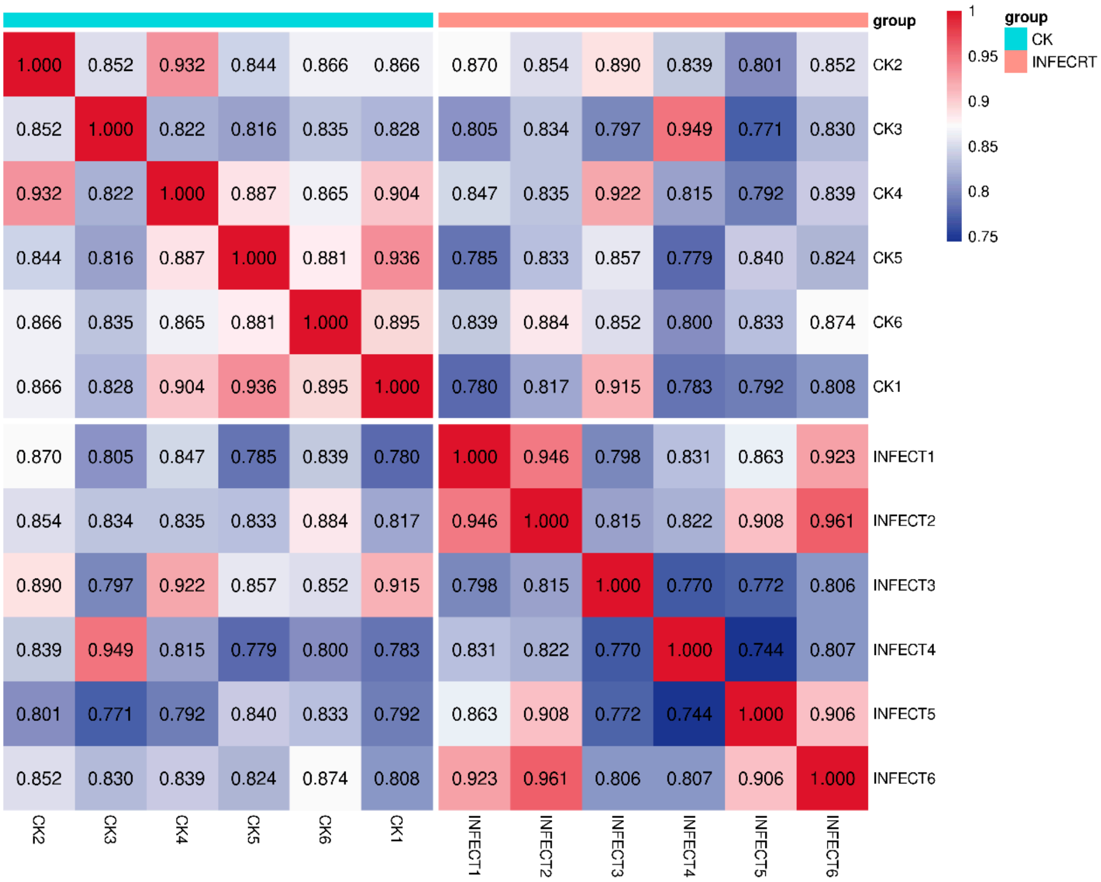

Figure S3. Pearson correlation heatmap of metabolite profiles between healthy (CK) and T. aurantii-infested (INFECT) tea leaf samples.

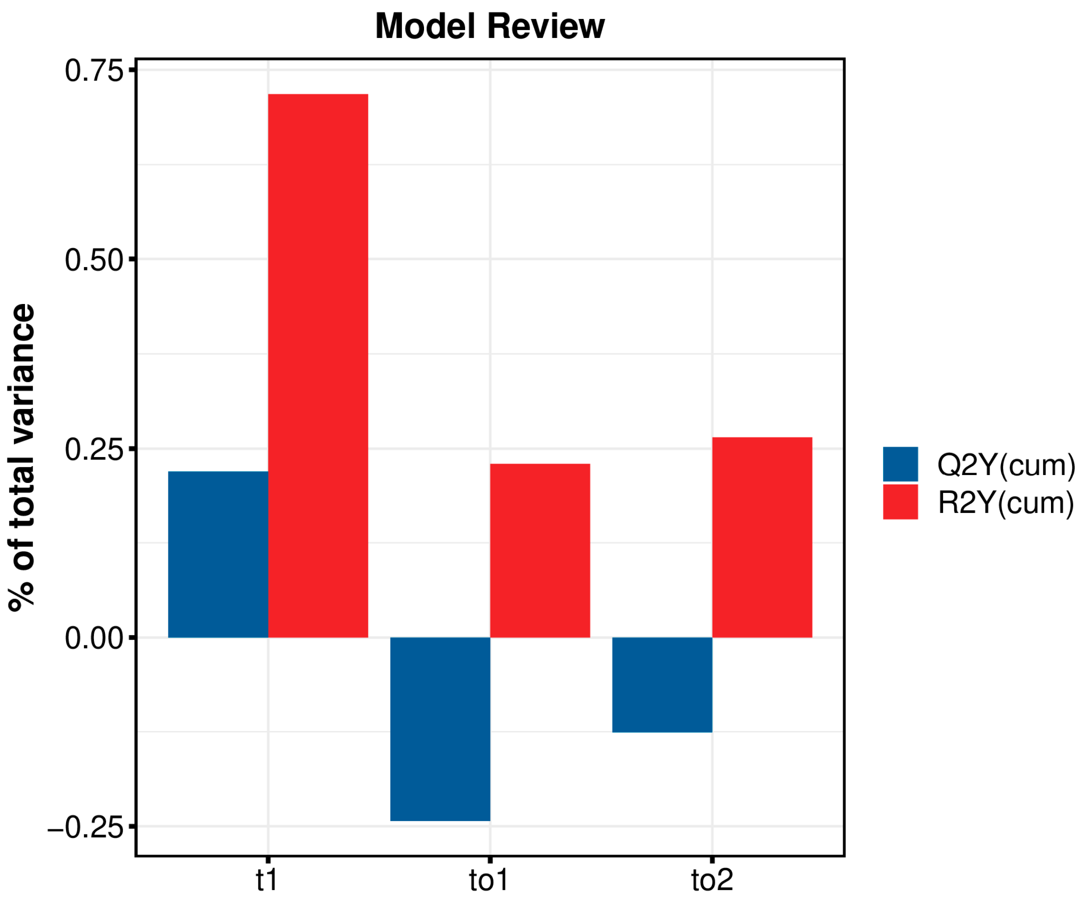

Figure S4. OPLS-DA model components and cumulative explained variances for the comparison between healthy (CK) and *T. aurantii*-infested (INFECT) tea leaves.

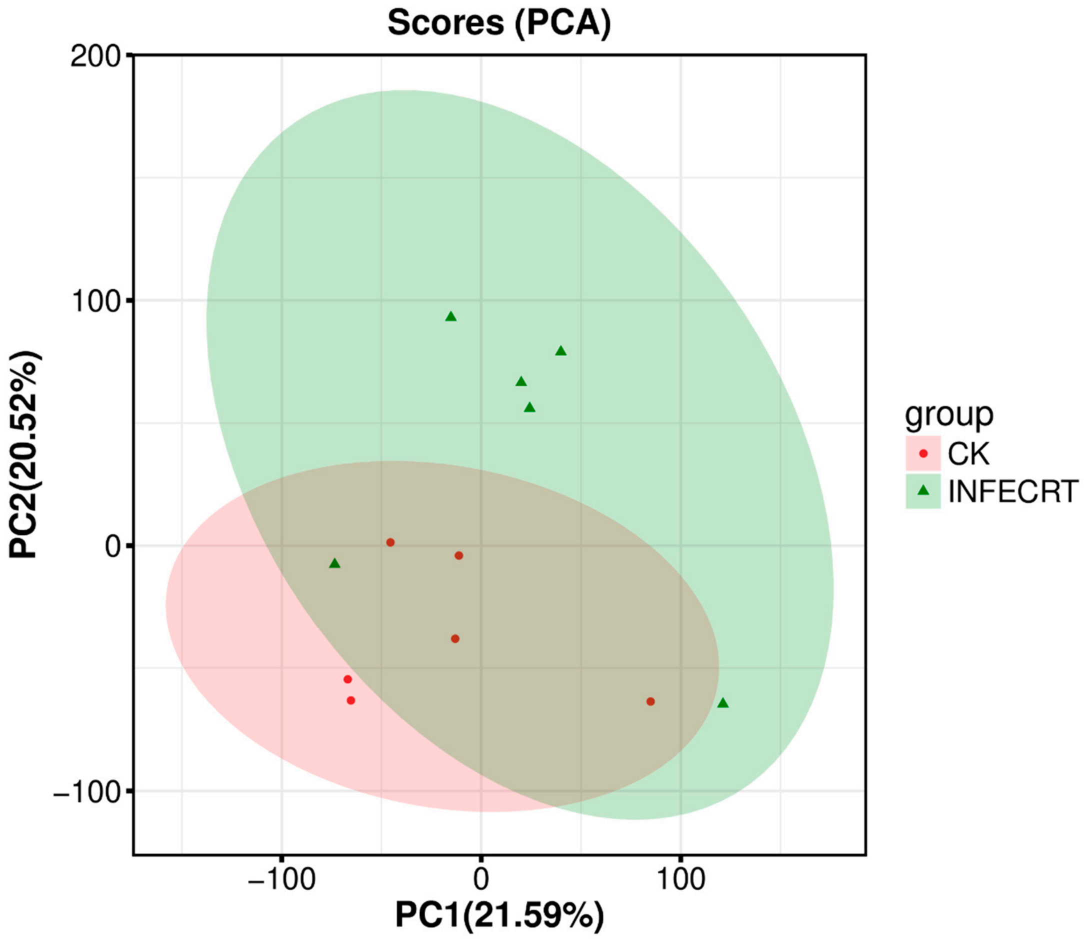

Figure S5. PCA score plot of metabolite profiles from healthy (CK) and *T. aurantii*-infested (INFECT) tea leaves.

Table S1. Significantly enriched bacterial taxa in each sample group identified by LEfSe analysis (LDA score > 4, Wilcoxon rank-sum test, $p < 0.05$).

Group	Biomarker	LDA_ score	P_value
Health leaf	k__Bacteria.p__Proteobacteria.c__Alphaproteobacteria.o__Rhizobiales.f__Beijerinckiaceae.g__Methylobacterium_Methylorubrum.s__Methylobacterium_brachiatum	4.99	0.0147
	k__Bacteria.p__Proteobacteria.c__Gammaproteobacteria.o__Burkholderiales.f__Comamonadaceae.g__Paucibacter	5.1	0.0047
	k__Bacteria.p__Proteobacteria.c__Gammaproteobacteria.o__Burkholderiales.f__Oxalobacteraceae.g__Massilia.s__Massilia_putida	4.44	0.0206
	k__Bacteria.p__Actinobacteriota.c__Actinobacteria.o__Micrococcales.f__Microbacteriaceae.g__Microbacterium.s__Microbacterium_testaceum	4.52	0.0449
	k__Bacteria.p__Proteobacteria.c__Alphaproteobacteria.o__Rhizobiales.f__Beijerinckiaceae.g__Methylobacterium_Methylorubrum.s__Methylobacterium_komagatae	4.2	0.0196
	k__Bacteria.p__Proteobacteria.c__Alphaproteobacteria.o__Rhizobiales.f__Beijerinckiaceae.g__Methylobacterium_Methylorubrum.s__Methylobacterium_aquaticum	4.35	0.0449
	k__Bacteria.p__Proteobacteria.c__Gammaproteobacteria.o__Burkholderiales.f__Comamonadaceae	5.06	0.0051
	k__Bacteria.p__Proteobacteria.c__Gammaproteobacteria.o__Enterobacterales.f__Erwiniaceae	4.63	0.0076
	k__Bacteria.p__Proteobacteria.c__Gammaproteobacteria.o__Burkholderiales	5.14	0.0168
Infected leaf	k__Bacteria.p__Proteobacteria.c__Gammaproteobacteria.o__Enterobacterales.f__Yersiniaceae.g__Serratia.s__Serratia_symbiotica	4.84	0.0048
	k__Bacteria.p__Proteobacteria.c__Alphaproteobacteria.o__Sphingomonadales.f__Sphingomonadaceae.g__Sphingomonas.s__Sphingomonas_sanguinis	4.89	0.0048
	k__Bacteria.p__Proteobacteria.c__Gammaproteobacteria.o__Enterobacterales.f__Erwiniaceae.g__Izhakiella.s__Dickeya_chrysanthemi	4.62	0.0074
	k__Bacteria.p__Proteobacteria.c__Alphaproteobacteria.o__Sphingomonadales.f__Sphingomonadaceae.g__Sphingomonas	4.98	0.0049
	k__Bacteria.p__Proteobacteria.c__Gammaproteobacteria.o__Enterobacterales.f__Yersiniaceae	4.83	0.0048

aphid	k__Bacteria.p__Proteobacteria.c__Gammaproteobacteria.o__Staphylococcales.f__Staphylococcaceae.g__Staphylococcus.s__Staphylococcus_xylosus	4	0.0125
	k__Bacteria.p__Proteobacteria.c__Gammaproteobacteria.o__Enterobacterales.f__Morganellaceae.g__Arsenophonus	5.24	0.0065
	k__Bacteria.p__Proteobacteria	5.48	0.0221
	k__Bacteria.p__Proteobacteria.c__Gammaproteobacteria.o__Enterobacterales.f__Morganellaceae.g__Candidatus_Hamiltonella.s__Candidatus_Hamiltonella_defensa	4.85	0.0096
	k__Bacteria.p__Proteobacteria.c__Alphaproteobacteria.o__Rickettsiales.f__Anaplasmataceae.g__Wolbachia.s__Wolbachia_endosymbiont_of_Drosophila_simulans	5	0.04
	k__Bacteria.p__Firmicutes.c__Bacilli.o__Staphylococcales	4.18	0.014
soil	k__Bacteria.p__Acidobacteriota	4.89	0.0124
	k__Bacteria.p__Bacteroidota	4.83	0.0196
	k__Bacteria.p__Chloroflexi	4.08	0.0048
	k__Bacteria.p__Verrucomicrobiota	4.37	0.0051
	k__Bacteria.p__Bacteroidota.c__Bacteroidia.o__Flavobacteriales.f__Flavobacteriaceae.g__Flavobacterium.s__Flavobacterium_alvei	4.18	0.0022
	k__Bacteria.p__Acidobacteriota.c__Acidobacteriae.o__Acidobacteriales	4.48	0.0192
	k__Bacteria.p__Chloroflexi.c__Ktedonobacteria	4.14	0.0022
	k__Bacteria.p__Patescibacteria	4.28	0.0022
